# Supplementary figures and images for: In Vivo Microevolutionary Analysis of a Fatal Case of Rhinofacial and Disseminated Mycosis Due to Azole-Drug-Resistant Candida Species
Source: J Fungi (Basel). 2023 Aug 2;9(8):815. doi: 10.3390/jof9080815 (PMC10455694; doi:10.3390/jof9080815)

Reads number

2015\_Ct 1

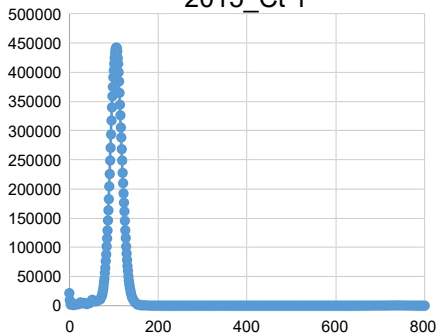

2015\_Ct 2

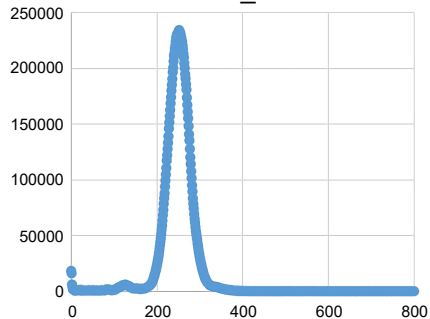

2016\_Ct 1

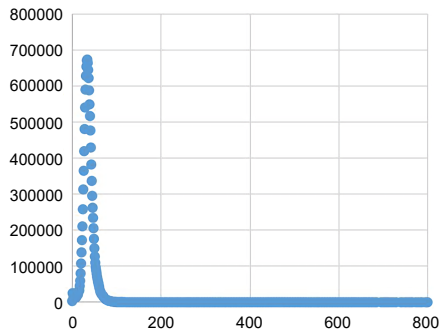

2016\_Ct 2

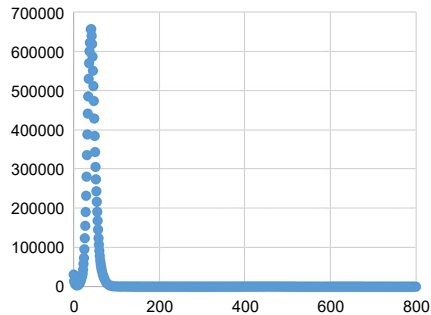

2016\_Ct 3

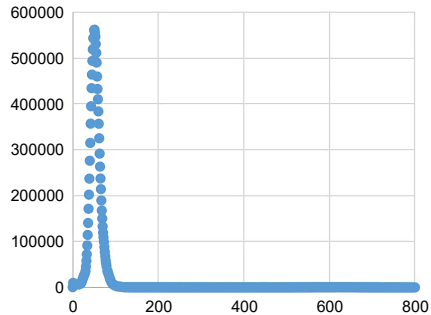

Depth

Supplement: Supplementary file 1 [file jof-09-00815-s001.zip › Supplementary_files/Supplementary_Fig_1.pdf]

2015\_Ct 1

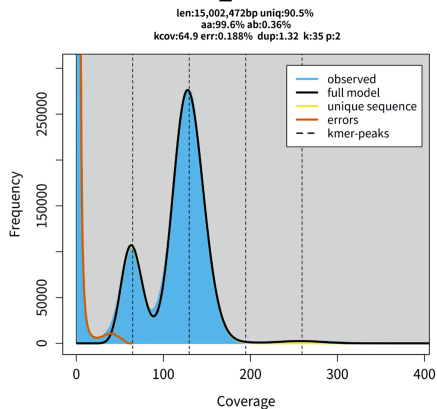

2015\_Ct 2

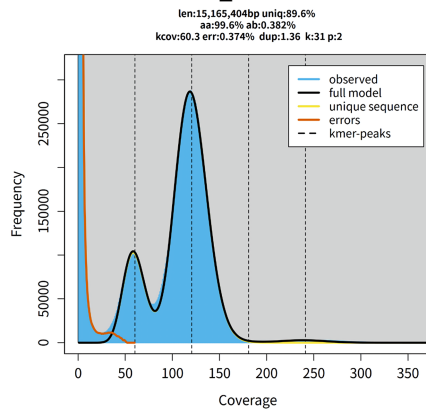

2016\_Ct 1

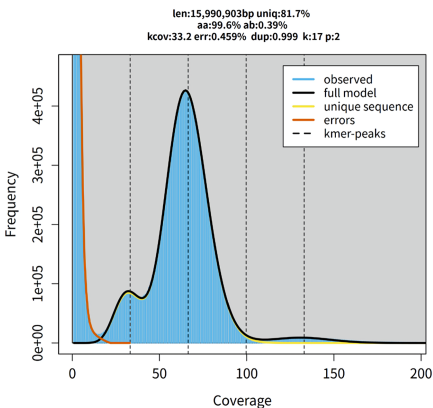

2016\_Ct 2

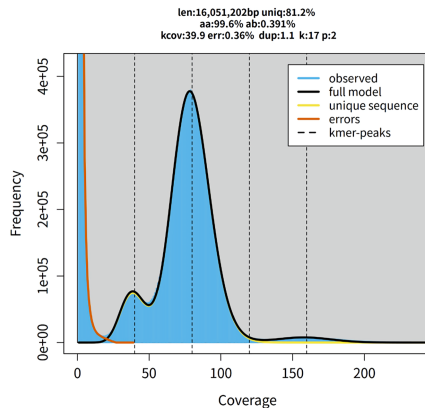

2016\_Ct 3

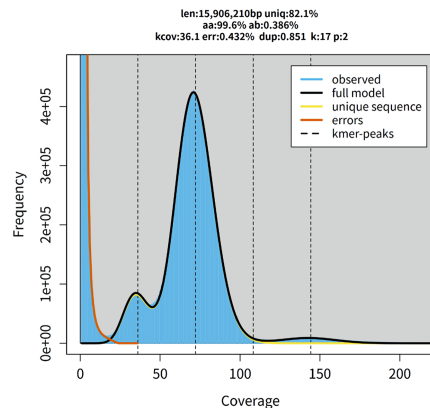

Supplement: Supplementary file 1 [file jof-09-00815-s001.zip › Supplementary_files/Supplementary_Fig_2.pdf]
