# Supplementary material for: In Vivo Microevolutionary Analysis of a Fatal Case of Rhinofacial and Disseminated Mycosis Due to Azole-Drug-Resistant Candida Species
Source: J Fungi (Basel). 2023 Aug 2;9(8):815. doi: 10.3390/jof9080815 (PMC10455694; doi:10.3390/jof9080815)
Supplement: Supplementary file 1 [file Supplementary_Fig_3.pdf › Supplementary_files/Supplementary_Fig_3.pdf]

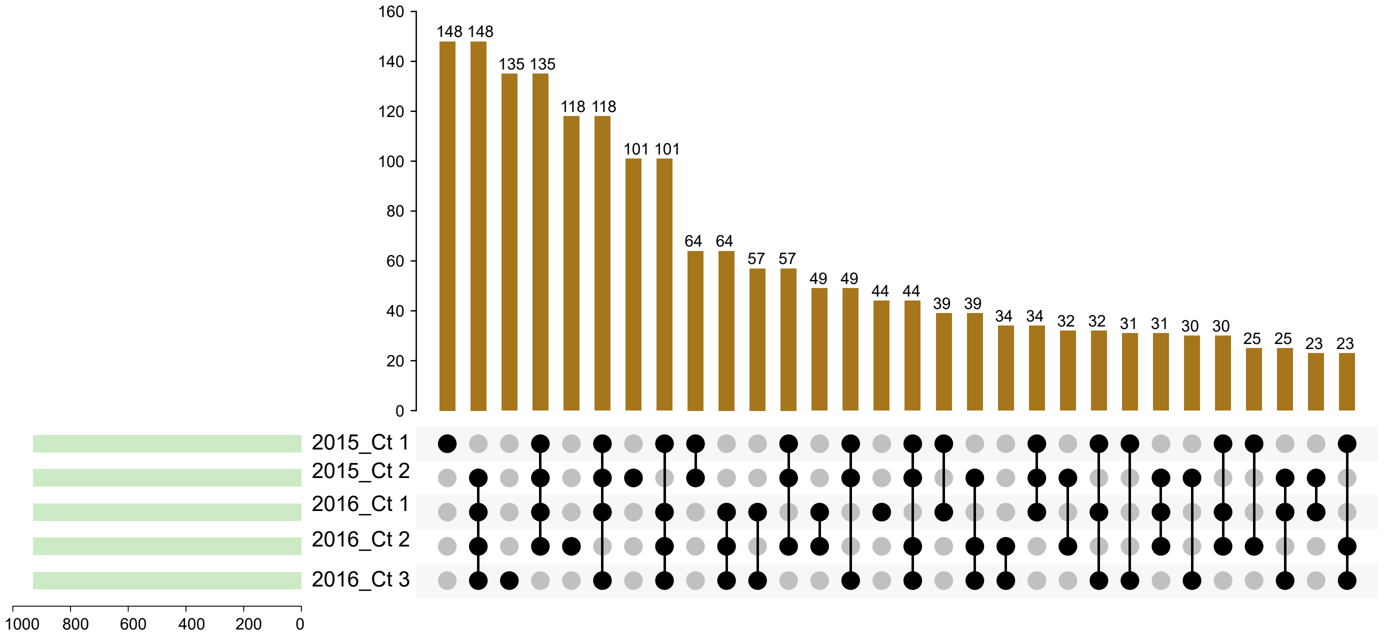

160
140
120
100
80
60
40
20
0
148 148 135 135 118 118 101 101 64 64 57 57 49 49 44 44 39 39 34 34 32 32 31 31 30 30 25 25 23 23
2015_Ct 1
2015_Ct 2
2016_Ct 1
2016_Ct 2
2016_Ct 3
1000 800 600 400 200 0
